# Inverse design with deep generative models: next step in materials discovery

**DOI:** 10.1093/nsr/nwac111

**Published:** 2022-06-11

**Authors:** Shuaihua Lu, Qionghua Zhou, Xinyu Chen, Zhilong Song, Jinlan Wang

**Affiliations:** School of Physics, Southeast University, China; School of Physics, Southeast University, China; School of Physics, Southeast University, China; School of Physics, Southeast University, China; School of Physics, Southeast University, China

## Abstract

Data-driven inverse design for inorganic functional materials is a rapidly emerging field, which aims to automatically design innovative materials with target properties and to enable property-to-structure material discovery.

In the past few years, machine-learning (ML) techniques have been extensively applied in material discovery. Such techniques are applied to minimize the computationally or experimentally expensive costs of the research process, greatly reducing overall design time [1]. Typically, ML algorithms are combined with traditional methods like first-principle calculations to accelerate the optimization of compositions on the known crystal structures (elemental substitution) in the database or literature, or to search for new configurations of a fixed chemical composition. The basic idea of this forward method is to predict the properties or new configurations of materials based on both domain knowledge and existing material data. Despite great progress, there remain several fundamental challenges. One is how to explore infinite chemical space towards the target region through the optimal path; another is how to rapidly and accurately develop materials with both stability and optimal properties [2,3]. Many advanced ML algorithms are widely used, including active learning and transfer learning, which enable the exploration of chemical space more efficiently. However, these methods have not yet been able to screen and evaluate all possible compounds in space, no matter how much computing power is available or how efficient the method is. More critically, it may be impossible to find materials with better performance based on known materials.

Inverse design, which refers to property-to-structure, is an emerging and different approach to finding compounds with desired properties [4]. Traditional ML-aided materials design involves directly predicting the properties of candidates in the entire chemical space. Then large-scale material screening is performed to search for promising materials with target properties. However, inverse design is different from the ML-aided global searching of known chemical space. It continues to generate qualified compounds along the optimal path, which brings new compounds with desired properties. Currently, two main techniques are being developed to achieve the goal of inverse design. One way to expedite the brute-force search for an optimal material is to perform global optimization in the chemical space [5]. In this case, methods like gradient descent are widely used and work well for energy. It is challenging to find new crystal structures with optimized properties as the physical properties might be discrete.

The data-driven generative model is another promising inverse design strategy (Fig. 1). In detail, the desired properties are firstly defined, and then materials with these properties are generated in an inverse way using generative models. In theory, a generative model attempts to build a map between chemical space—which is obtained by learning a large amount of known data via a deep neural network—and real space. This map can be further enhanced with additional information (physical properties) to condition or bias the generative process. In practice, in addition to the required physical properties, the materials generated also have good stability or synthetic possibilities. Therefore, inverse design needs to consider both the physical properties and formability or stability of potential materials in the same model, which can be done by building an appropriate loss function.

**Figure 1.**
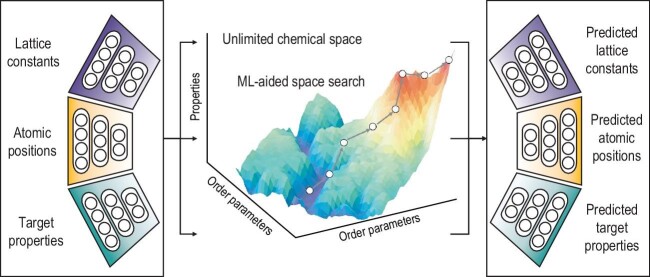
Schematic diagram of the inverse design process.

Currently, generative models, such as vibrational autoencoders (VAEs), generative adversarial networks (GANs), reinforcement learning and recurrent neural networks, are regarded as ideal inverse design methods to address the above computational difficulty with regard to searching [3]. These algorithms have been widely applied in many fields, such as molecular synthesis and drug discovery [6]. For example, Baekjun Kim *et al.* proposed a GAN to generate 121 novel crystalline porous materials based on over 30 000 known zeolites [7]. Niklas W.A. Gebauer *et al.* proposed a conditional generative model for generating 3D organic molecules with specific chemical properties and structures [8]. Beyond theory, advances of inverse design in experiments are even more revolutionary. Connor W. Coley *et al.* built an automated synthesis platform that combines artificial-intelligence-planning synthesis routes and robotic execution [9]. The platform trains ML algorithms based on reactions in American patents and the Reaxys database.

Nevertheless, the application of inverse design to solid-state materials is just getting started and faces many challenges. First, differently from organic molecule databases, inorganic material databases only have, roughly, hundreds of thousands of compounds available, with limited structural diversity. Even worse, materials with specific properties, such as ferromagnetism, are rather scarce. This often leads to the incomplete training of ML models, and it may not be possible to generate meaningful new compounds from existing materials. Second, invertible and invariance representations for periodic crystal structures are lacking. If materials can be reversibly represented, the generative model can transform the mathematical output of a neural network into a crystal structure automatically.

Several attempts have been made to address the aforementioned challenges using generative models. In the early stages of inverse design, Noh *et al.* developed a generative framework using hierarchical two-step VAE models to discover new vanadium oxide materials [10]. The scheme is achieved by utilizing three-dimensional image-based invertible input representation for crystal structures for both cell and basis information. But the process of decoding images back to atom types and coordinates often results in low validity, and the models are not rotationally invariant. Kim *et al.* adopted a different strategy to make the generative model reversible and invariant [11]. Crystal structures are converted to a representation that is inversion-free based on a set of atomic coordinates and cell parameters, and data augmentation is applied to initial data sets. Then, a Wasserstein GAN is applied to generate new Mg-Mn-O ternary compounds to find potential semiconductors with reasonable stability in an aqueous environment. These results represent a significant step toward inverse design using generative models. However, the generative models are limited to a specific composition or crystal structure. Realizing the general aspect of inverse design has a more practical significance. For this reason, Yao *et al.* proposed an automated nanoporous material discovery platform powered by a supramolecular VAE for the generative design of reticular materials. The automated design process contains a class of metal-organic framework structures [12]. Ren *et al.* presented a framework capable of general inverse design where the designed materials are of various chemistries and structures. The model utilizes a generalized invertible representation that encodes crystals in both real and reciprocal space, and generates a property-structured latent space from a VAE [13].

To sum up, inverse design has shown great potential in physics, chemistry and materials science. Nevertheless, many challenges still restrict its further development. In detail, in order to be used in more practical applications, inverse design requires further technological innovations in data accumulation, material representations and generation models. Firstly, in terms of data, the published scientific literature is another potential source of material data in addition to the existing material database and high-throughput calculations/experiments. So much data in the literature can be extracted using methods such as natural language processing. Secondly, crystal-graph-based techniques have shown great potential in ML direct prediction of material properties [14]. Thus, an invertible and invariant crystal-graph-based representation would be beneficial to the development of more robust generative models for inorganic materials. Thirdly, in addition to basic generative models like GANs, developing new generative models for small data sets is critical. For example, active learning can prioritize the data that need to be labelled in order to have the highest impact on ML model training. And transfer learning can store knowledge gained while solving one problem and apply it to a different but related problem. These algorithms, as powerful tools, have been applied to solve the problem of material data scarcity [15]. In combination with generative models, they may be a potential way to further promote the application of inverse design. In addition to the above three technical challenges, current inverse design frameworks are still inadequate, and developing more advanced and general frameworks, which are applicable to an unlimited number of compositions and complex properties, remains a grand challenge. Beyond theory, closed-loop approaches for material discovery using generative-model-based inverse design will be capable of navigating and searching chemical space quickly, efficiently and, importantly, without bias. In any case, the extensive application of inverse design in materials science will further change the research paradigm of materials science, and bring material design into the age of automation.
